# Hidden costs to building foundations due to sea level rise in a changing climate

**DOI:** 10.1038/s41598-022-18467-3

**Published:** 2022-08-18

**Authors:** Mohamed A. Abdelhafez, Bruce Ellingwood, Hussam Mahmoud

**Affiliations:** Department of Civil and Environmental Engineering, Colorado State University, Fort Collins, CO USA

**Keywords:** Civil engineering, Natural hazards

## Abstract

Coastal civil infrastructure is vulnerable to the effects of climate change. Hurricane storm surge and coastal flooding can cause significant hydrostatic and hydrodynamic loads on structures while saltwater intrusion (SWI) may lead to deterioration of foundations. The effects of saltwater intrusion due to Sea Level Rise (SLR) on the foundations of buildings and other civil infrastructure is poorly understood. Such damages may not be detected in a timely fashion nor be insured, leading to significant and unanticipated expenses for building owners. In this study, we evaluate the impact of SWI due to various SLR scenarios on the corrosion of reinforcement in foundations of nearly 137,000 residential buildings in low-lying areas surrounding Mobile Bay, AL. We find that the potential for costly damage is significant. Under an extreme SLR scenario, the annual expected repair costs for the foundations of the studied homes may reach as much as US$90 million by 2100.

## Introduction

Sea level rise (SLR) is a significant effect of a changing climate^1,2^. Ecosystems, human settlement, and vital community services in low-lying coastal plains are all threatened by SLR, which impacts about half a billion people globally^3^. Flooding of coastal lowlands^4^, as well as damage to marine ecosystems^5^, the built environment^6,7^, and marine transportation systems (seaports)^8,9^ are all consequences of SLR. Furthermore, in roughly 20% of the Earth’s arable land, including coastal regions of the United States, soil salinity is a problem^10^. Soil salinity may be ascribed to many factors in addition to SLR, including natural buildup, irrigation, land aridity and human activity. Salts are often brought into the soil via a rising water table or sub-surface movement of water within 1–2 m of the soil surface^11^. According to current estimates, the rise in the groundwater table in coastal areas due to rising sea levels is approximately proportional to the rise in sea level^12,13^.

Excessive salinity in ground water may cause major problems for local agriculture, foundations of structural systems, roads, bridges, phone lines, water systems and sewers^11^. For example, some underground utilities (electric, cable, and telephone) in New Orleans were severely corroded due to the effects of seawater inundation from Hurricane Katrina^14^. Saha and Eckelman^15^ concluded that by 2055, existing concrete buildings in Boston, MA within 10 km of the coastline might experience chloride intrusion exceeding code-recommended reinforcing bar cover thicknesses. Many other studies have considered the impact of de-icing salts on concrete bridge decks^16–20^. However, the authors are unaware of any previous studies that have examined potential deterioration of reinforced concrete building foundations in coastal regions due to saltwater intrusion (SWI) brought about by SLR due to climate change.

In a maritime environment, chloride-induced corrosion of reinforcing bars is the main cause of degradation in reinforced concrete components^21^. Corrosion initiates when a certain concentration of chlorides accumulates on the surface of the reinforcement bars in the concrete element, which is often due to insufficient concrete cover^22^, as noted in the “Methods” section. Subsequent corrosion of the reinforcement leads to a decrease in structural capacity and, in addition, is usually accompanied by cracking and spalling due to the expansive nature of the corrosion process. If the concrete element is easily accessible, periodic inspections and maintenance often will uncover the problem and permit timely and appropriate repair or rehabilitation of damaged structural components. On the other hand, foundation walls and slabs frequently are partially or completely inaccessible for periodic inspection, in which case their deterioration may not be detected until it is manifested through damage to the superstructure. In such instances, repair or rehabilitation may be very costly. A similar problem may exist in regions of the United States (particularly in the southern Great Plains region) where expansive soils are prevalent. In such regions, buildings are often constructed as slab-on-grade to minimize the need for inspection and the likelihood of costly foundation deterioration.

**Figure 1:**
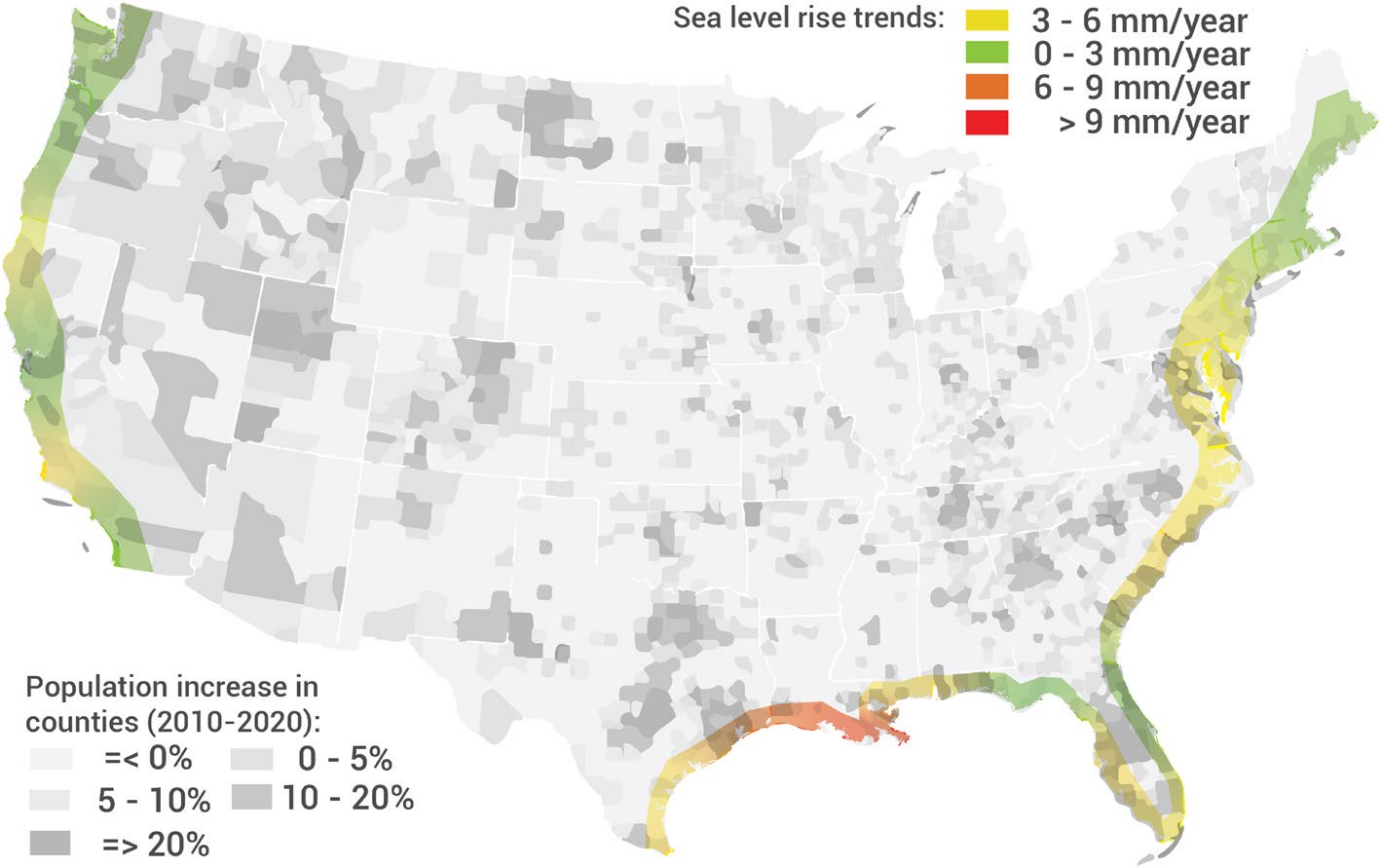
Relative sea-level trends at coastal counties in the US^23^ overlapping with the population growth in the last 10 years^24,25^. The software used to develop the map in this figure is QGIS 3.16 (https://qgis.org).

Figure 1 shows regions of the coastal US that are especially susceptible to sea level rise due to climate change. The Southeast and Gulf Coast are also regions of significant population and economic growth. The coastal region extending from Boston, MA in a southwesterly direction toward Pensacola, FL supports significant industrial infrastructure, with three major seaports and many of the nation’s largest petroleum refineries, chemical and energy corporations, and aerospace and defense industries located on or close to the coast. Construction brought on by urbanization in these regions will be impacted by increases in the ground water table which exposes foundation systems to saltwater intrusion and corrosion.

In this study, we consider the impact of a changing climate on civil infrastructure from a new perspective: the negative impact of SLR on the foundations of buildings and other civil infrastructure caused by SWI. We consider the probable damage to nearly 137,000 residential buildings in low-lying regions within Mobile County, Alabama caused by the influence of the SWI resulting from SLR scenarios on the corrosion of their foundations through the remainder of the twenty-first century. We outline the conceptual framework of our analysis to help readers quickly grasp the most important ideas. The subsequent “Methods” section provides further details on our analytical approaches, while the “Supplementary Information” (SI) goes into greater depth regarding the datasets, figures, and tables that were used in the analysis. Our results show that buildings located 5–10 km away from the shoreline are the most vulnerable under plausible SWI scenarios. We introduce three example risk mitigation planning strategies and illustrate their relative advantages using a life-cycle approach to guide public policymakers in the future. Additional findings of the mitigating techniques are described in the SI S2 and S3.

## Analysis of SWI: conceptual framework

A variety of non-compatible datasets are required in order to evaluate coastal buildings for SWI due to SLR. These datasets include the buildings inventory, the mechanism of SWI, variations of soil types, and fluctuations of the ground water depths (GWD) that is measured from ground level at each of Mobile County's 4719 wells, many of which have been monitored over the past century. Following a summary of these datasets, a procedure is proposed to evaluate buildings' foundations for deterioration.

## Description of building inventory in Mobile County

**Figure 2:**
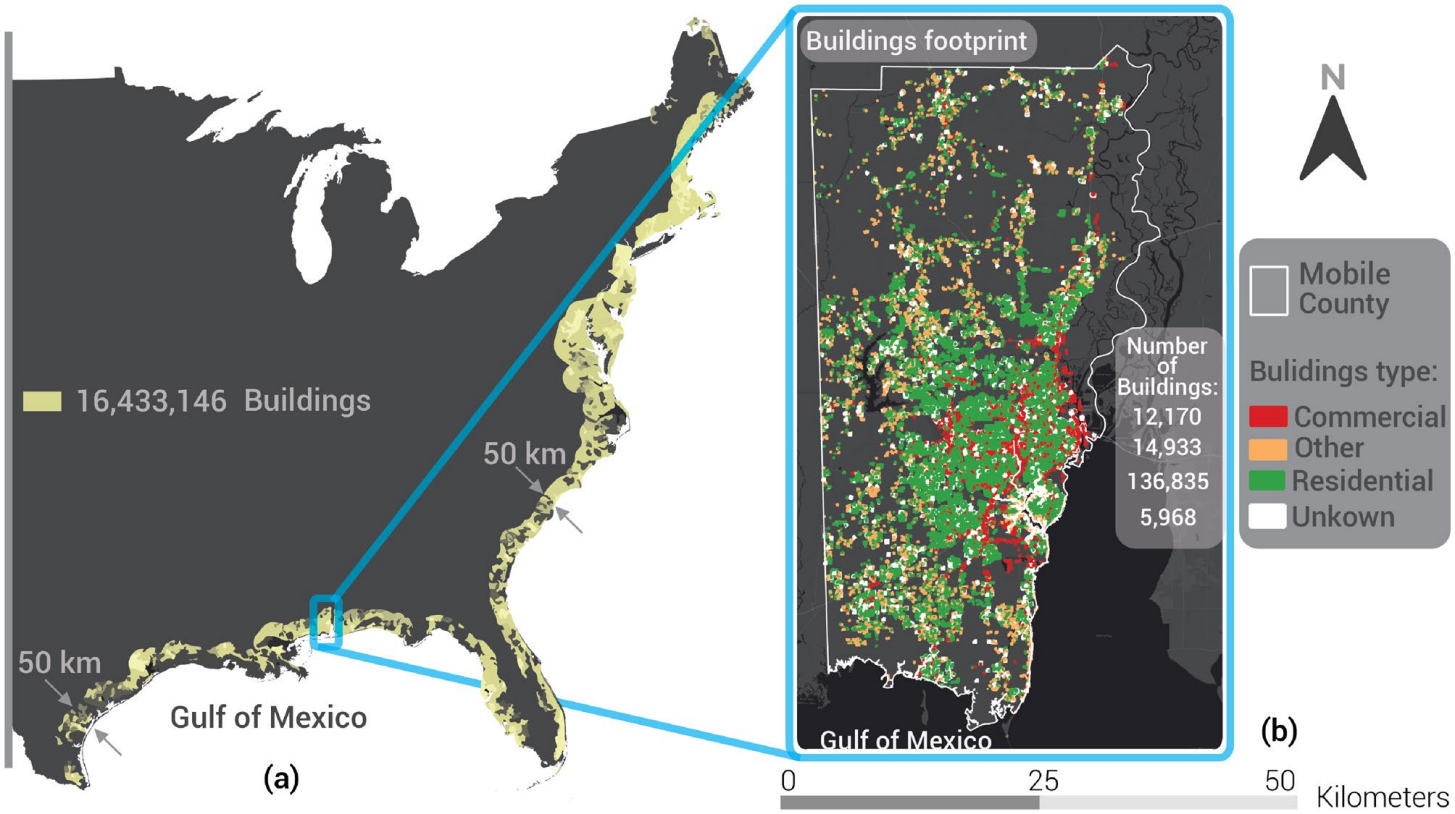
(**a**) Number of buildings located from the shoreline to 50 km inland and (**b**) the building types in Mobile County, AL. The software used to develop the maps in this figure is QGIS 3.16 (https://qgis.org).

The density of urban development in coastal regions of the U.S. and the building inventory in Mobile County, AL were assembled from Microsoft Footprint^26^ and are illustrated in Fig. 2. Based on previous studies^27–29^, the extent of SWI can vary from 5 up to 50 km inland from the Gulf of Mexico (GoM) and the east coast (EC) of the US Approximately 16.4 million of the 47.7 million buildings located in coastal states of the GoM and EC in the US are located within 50 km of the shoreline (Fig. 2a) and may be vulnerable to SWI due to climate change. Microsoft's footprint data does not provide information about each building that is sufficiently detailed to assess its performance from an engineering perspective (i.e., building type, construction material, number of stories, and foundation depth). To overcome these limitations, we utilized a dataset obtained from ATTOM Data Solutions^30^, which matches nearly 300 building attributes to 169,906 buildings in Mobile County, AL. ATTOM data enables us to distinguish between residential, commercial, and other constructions. Using the data, we can also determine how many floors each building has and when it was constructed. Figure 2b identifies common buildings occupancies located in Mobile County using the ATTOM datasets. Some attributes used in our analysis are explained in the SI S1, while other necessary features (e.g., foundation depth) were calculated using soil type data (Supplementary Fig. S2.1).

## Saltwater intrusion scenarios

The intrusion of seawater is particularly common in coastal aquifers, where water tables are below sea level, but it can also occur in some coastal aquifers when groundwater is lowered by pumping^31^. Investigations of seawater intrusion have been conducted at various locations along the Atlantic and Gulf coasts^32^. While no previous studies on SWI in Mobile County could be found, Baldwin County, which is located on the east side of Mobile Bay, has similar soil characteristics as Mobile County, as shown in Supplementary Fig. S2.1. In addition, ground elevations in Mobile County are similar to the elevations in Baldwin County, as shown in Supplementary Fig. S2.2. In fact, SWI was first noted in Baldwin’s coastal aquifers in 1985, when a rise in salinity of shallow groundwater in coastal regions was reported as a result of hurricanes in the Gulf of Mexico caused by sea water spray and flooding^32^. A more recent study showed that salinity levels in wells have increased in the coastal zones of Baldwin and Mobile counties due to saltwater intrusion into the aquifers^33^, to the point where salinity of almost 17.5 parts per thousand (ppt) or (g/l) extended nearly 10 km inland in Baldwin County. The Alabama Water Watch website^34^ provides salinity measures in rivers and coastal shorelines for Mobile and Baldwin counties, which show that salinity concentrations range from 5 to 20 ppt and extend 0.5–10 km inland from Mobile Bay. Thus, SWI studies in Baldwin are used to identify SWI scenarios for Mobile County and to estimate the impact of different salinity concentrations on the corrosion of reinforcement in building foundations. Based on the previous study^33^, the information found on the Alabama water watch website^34^, and the fact that chloride content represents 55% of salinity content^35^, we consider four SWI scenarios in the groundwater of Mobile County, which are summarized in Table 1. Those within 10 km of the shoreline are covered by SW1, whereas SW4 covers buildings further inland.

**Table 1:** The SWI scenarios used in this study.

Scenarios	Salinity (ppt) (g/l)	Chloride (ppt) (g/l)
SW1	20	11
SW2	15	8.25
SW3	10	5.5
SW4	5	2.75

## Framework for assessing degradation of building foundations

**Figure 3:**
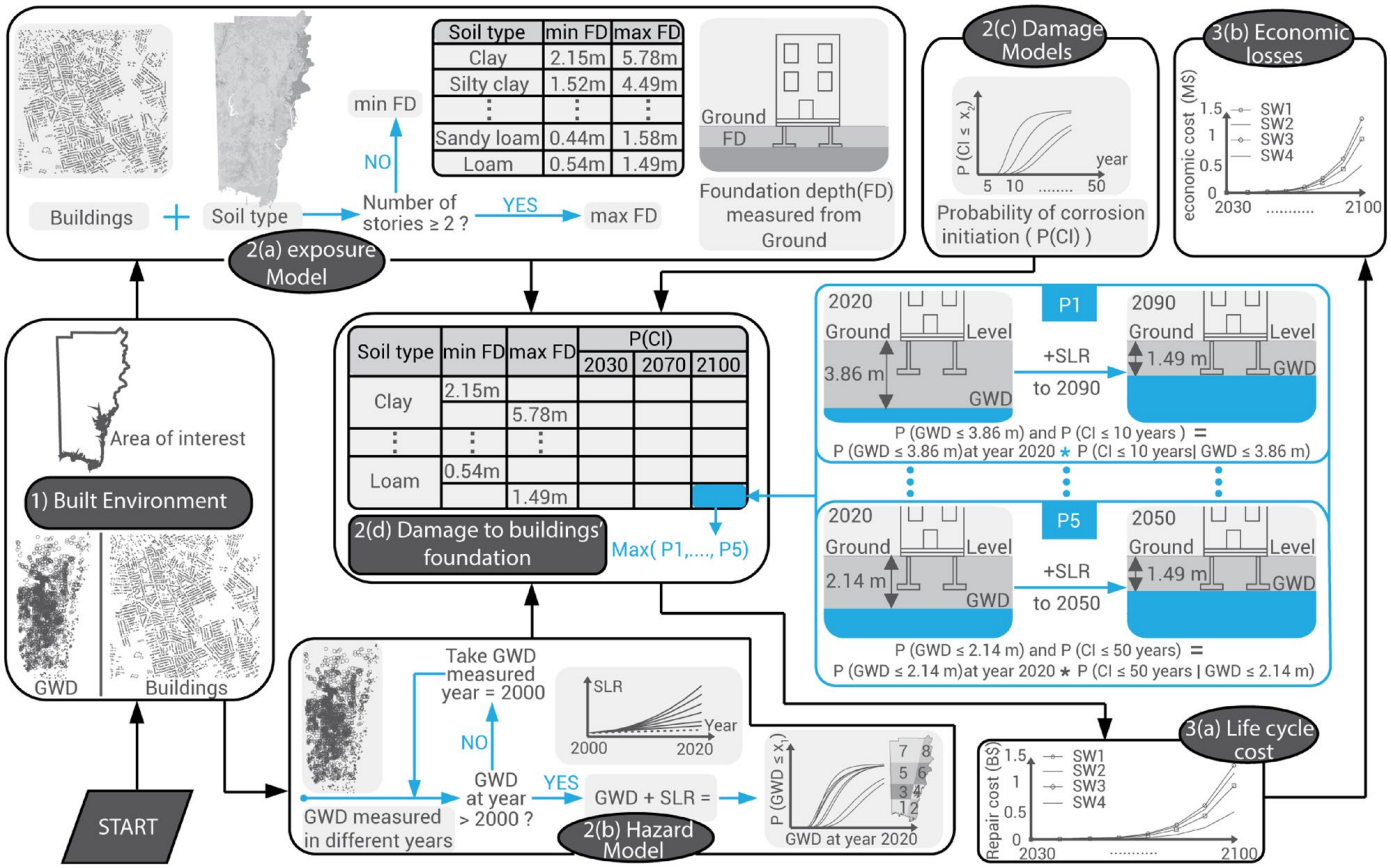
Building foundations deterioration assessment framework.

The deterioration of residential building foundations is modeled in three main steps, as shown in Fig. 3. Step 1 defines the built environment, in which the building inventory is assembled (summarized above and explained in detail in the SI S1) and GWD is estimated (details are provided in “Methods”). Step 2 determines the damage to the foundation in four sub-steps, which are explained in “Methods”. Step 2(a) explains the exposure to different SWI scenarios (reflected in the salinity of the groundwater) which relies mainly on the foundation depth (FD) of each residential building. Minimum and maximum values for the FD are calculated as outlined in Supplementary Table S2.1 and in “Methods”. Thus, we assign a minimum FD if the building has one story and maximum FD if a building has two or more stories. The hazard model (Step 2(b)) explains the rise of the GWD table due to the projected SLR scenario. As further illustrated in “Methods”, GWD is measured in different years. Because there was little SLR prior to the year 2000, we assumed that there was no change in GWD if it was measured prior to 2000. Conversely, if the measurement was after the year 2000, the rise of the GWD table for each well is assumed to track the SLR projection linearly^12,13^, starting from the year in which the measurement was taken. Step 2(c) deals with the uncertainties related to the probability of corrosion initiation (P(CI)), which is explained in detail in “Methods”.

Damage to the building foundation (Step 2(d)) explains how the probability of corrosion initiation, (P(CI)), is determined every 10 years from 2030 to 2100 using inputs from Steps 2(a), 2(b), and 2(c). The P(CI) is calculated every 10 years for each soil type by taking the maximum probability of corrosion initiation at a certain year (2030–2100), due to 10, 20, 30, 40, and 50 years of exposure to the chloride concentration obtained from SWI scenarios. For example, to calculate the probability of corrosion initiation for a multiple-story building located on loam soil, P1 (10 years chloride exposure) is defined as the probability that GWD is less than 3.86 m in 2020 and GWD is decreased to 1.49 m (max FD for loam soil) in 2090 using SLR projections. Probability of corrosion initiation, P5, is defined as the probability that GWD is less than 2.14 m in 2020 and GWD is decreased to 1.49 m in 2050, exposing the foundation to chloride for 50 years.

Step 3 determines the economic consequences of corrosion damage. Life cycle cost (Step 3(a)) shows the repair cost for the foundation due to the corrosion damage for each building (the output from 2(d)) every 10 years from 2030 to 2100, as explained in detail in “Methods”. Economic losses (3(b)) include the cost of repairing the building’s foundation, which usually requires 2–3 days on average^36^, and the cost incurred by residents who must relocate to temporary quarters during the period required for the foundation to be repaired.

## Damage and losses due to foundation reinforcement corrosion

**Figure 4:**
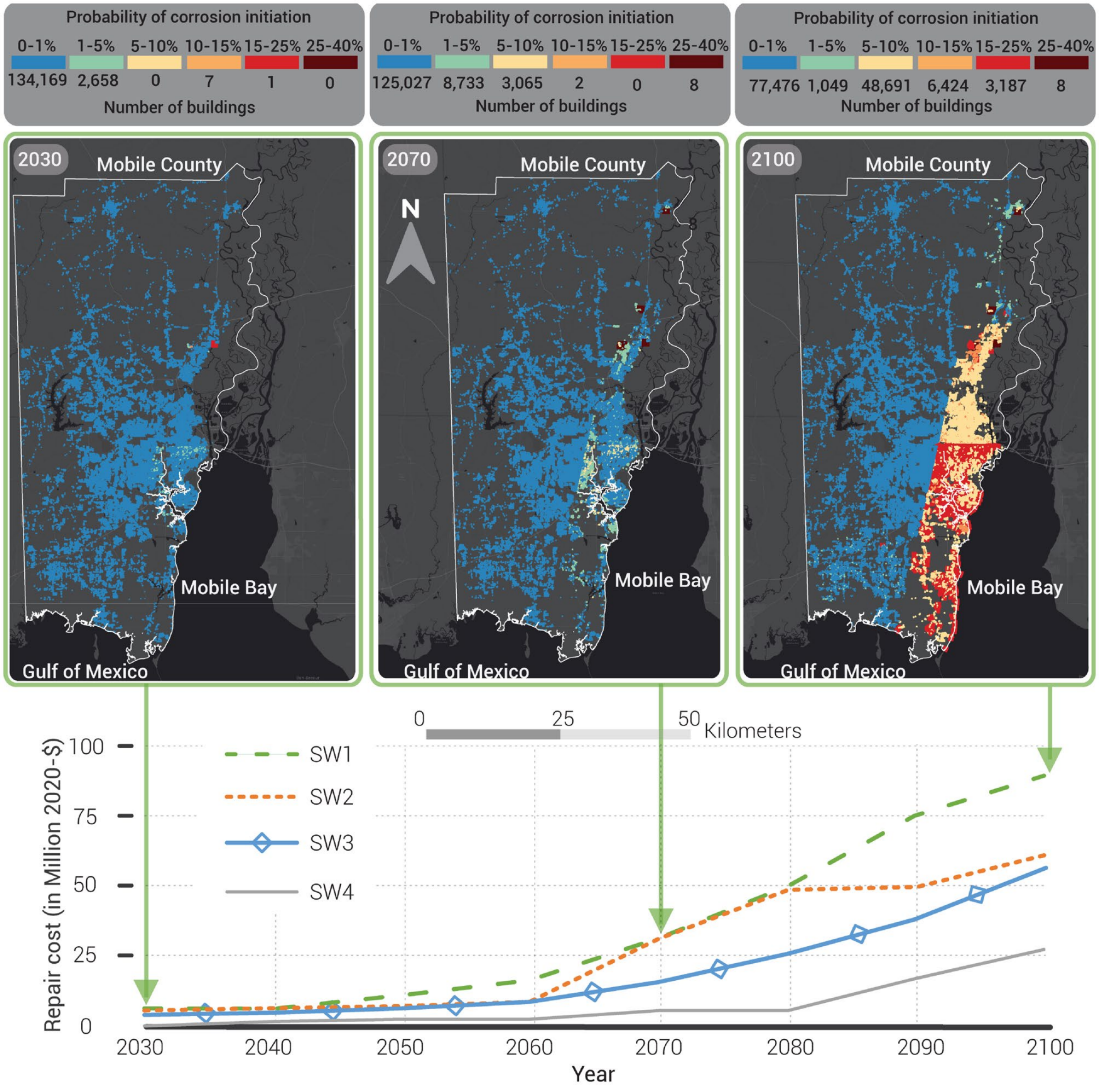
The repair cost in the absence of any mitigation under each scenario with the probability of corrosion initiation on the foundation of residential buildings under the SW1 scenario. The software used to develop the maps in this figure is QGIS 3.16 (https://qgis.org).

We assumed that the corrosion-related damage begins at the time at which corrosion initiates^37^ and that the probability of corrosion initiation equals the degree of damage due to corrosion. The probability of corrosion initiation from the four SWI scenarios revealed the sensitivity of building foundations to deterioration caused by SWI due to SLR from climate change. As shown in Fig. 4, high exposure of the foundation to chlorides (SW1) led to only minor corrosion (1–5%) in around 2% of all residential buildings by the year 2030. By 2070, the percentage of building foundations with minor corrosion (1–5%) increased to almost 7%. By 2100, 36% of the building foundations had a probability of corrosion initiation equal to 5–10% while 7% indicated probabilities in the range 10–25%. The projected SLR is the main reason why the number of corroded buildings is higher in year 2100 compared to year 2070, as explained in the “Methods” section. Only a few buildings had probabilities of corrosion initiation as high as 25–40% by the year 2070; those buildings are located on clay soil (FD is 4.07 m below ground level) or clay-loam soil (FD is 5.78 m below ground level) and are two or more stories in height. It should be noted that clay soil and clay-loam soil have the highest clay content compared to the other soil types, as shown in Supplementary Table S2.1. Such buildings will be at higher risk by 2070 than others, suggesting that inspection every 10 years might be required for those buildings located on such soils with high GWD. Inland buildings (Supplementary Fig. S2.3a, region 1) that are located on high clay content soil have a probability of corrosion initiation in range 1–5% and 5–10% due to SW3 (Supplementary Fig. S3.2a) and SW1 (Fig. 4) by 2100. However, inland buildings in regions 3, 5, and 7 showed very low corrosion initiation probability (0–1%), as can be seen in Fig. 4 and Supplementary Figs. S3.1 and S3.2. Soil types in region 4 are mainly loamy sand, sandy loam, and loam soils with high sand content, as shown in Supplementary Table S2.1. However, the buildings in region 4 were vulnerable to SWI due to a high GWD and number of stories more than two (deeper FD). These results indicate that the susceptibility of inland building foundations to SWI depends on the GWD and the soil type (Supplementary Fig. S2.1 and Supplementary Table S2.1). Most vulnerable buildings are located near the shoreline, with corrosion initiation probabilities of 15–25% by 2100, as shown in Fig. 4 (regions 2 and 4) and Supplementary Fig. S3.1 (region 4).

Figure 4 shows the total repair cost for each scenario at 10-year intervals, assuming no repairs were done previously. For example, the total repair cost discounted to the present value, (PV), as explained in “Methods”, would be around $50.35 million to repair the corrosion to the foundations in the year 2080 under the SW1 scenario, assuming no repairs were performed prior to 2080. Consequently, deferring inspection until 2080 would lead to higher repair costs ($90 million in 2020-$), as shown in year 2100. That can be noticed more clearly by the year 2100 in SW4, the lowest chloride exposure, which leads to similar repair costs to SW1 in the year 2070. As noted previously, repairing the building foundation would also require temporary accommodation for its residents. Census data^38^ indicated that the average household size of occupied housing units is 2.7, and the average population density in Mobile County is three persons per residential building. Moreover, 90% of the residential buildings in our dataset are one-story houses and the rest are two-story houses. Hence, we assume 3 to 4 residents live per house. An average hotel night in Mobile County would cost around $100 for a double bedroom. A temporary relocation of residents for two nights under SWI leads to an additional cost of $0.8 million, $1.12 million, and $2.39 million (in 2020-$) for the residents of 2,666, 11,808, and 59,359 houses by years 2030, 2070, and 2100.

## Risk mitigation strategies: periodic inspection and repairs using corrosion inhibitors

The preceding analysis has shown that rising sea levels and SWI represent a significant threat to the foundations of coastal buildings during the remainder of the twenty-first century. Beyond physical damages and economic losses to residential properties, the social impacts may involve losses in employment and outmigration of population^39,40^. The evolving nature of these hazards must be recognized in risk mitigation planning for future development and protection of existing coastal residential buildings. The optimal approach to risk mitigation planning depends on the nature of the community, which is identified by the nature of socio-economic institutions, tax base, educational level of the residents, etc., but is outside the scope of the current study. While this section focuses on residential building portfolios, the range of interruptions caused by SLR and SWI within the community should be included in any complete risk mitigation strategy. The following subsection illustrates three possible risk mitigation strategies, evaluated in terms of estimated life cycle cost. Periodic inspection is an obvious strategy for corrosion-related risk mitigation, one that has been used to mitigate damage to bridges^20,41^, so the risk mitigation strategies are built around that concept.

Since most residential buildings in Mobile County were constructed between 1980 and 2015, suppose that it is possible to inspect each vulnerable property in susceptible coastal regions (regions 1, 2, 4, 6 and 8 in Supplementary Fig. S2.3) every 10 years (option A), 20 years (option B), and 40 years (option C) during the remainder of the twenty-first century (80 years). As reinforcement corrosion is an expansive reaction, there is the possibility of the development of visual cracks on the concrete. Thus, we used a 10% threshold for the probability of corrosion initiation to indicate hairline cracks (0.05 mm)^42^, which the inspector can detect and which may prompt a request to repair the building foundation. We also assumed that corrosion-inhibiting materials would be used to decrease the corrosion initiation rate when a repair is implemented. Once an inhibitor is added to the mortar and applied after repairing the corroded bars in the foundations, the chloride diffusion coefficient is set equal to 32 mm^2^/year^43^ rather than the value in Supplementary Table S2.2. Moreover, the chloride threshold (Supplementary Table S2.2) is increased from 1 to 1.72 kg/m^3^^44^ if a surface-applied corrosion inhibitor has been used externally over the concrete surface of the foundation. For mitigation option A (every 10 years inspection), the inspections are assumed to commence in 2030, assuming 10 years of the foundation’s exposure to chloride from 2020. Moreover, we assumed for this strategy that only P1 (from (2-d) in Fig. 3) will be calculated since the inspectors will not allow corrosion to develop for more than 10 years. For mitigation option B (every 20 years inspection), the inspections are considered to start in 2040. In addition, we assumed for this strategy that both P1 (10 years exposure) and P2 (20 years exposure) from Fig. 3 will be calculated since the inspectors will not allow corrosion to grow more than 20 years. Mitigation option C (inspection every 40 years) calls for the inspection to occur only in the year 2060 and 2100. Because inspectors will not allow corrosion to grow for more than 40 years, probabilities P1 to P4 (Fig. 3) are required for option C. We assume in the three mitigation options that the residential buildings remain occupiable in the year 2100, leaving the question as to whether the buildings would remain occupied beyond this point open while noting that the average service life of a residential building in the US is on the order of 100 years^45^.

**Figure 5:**
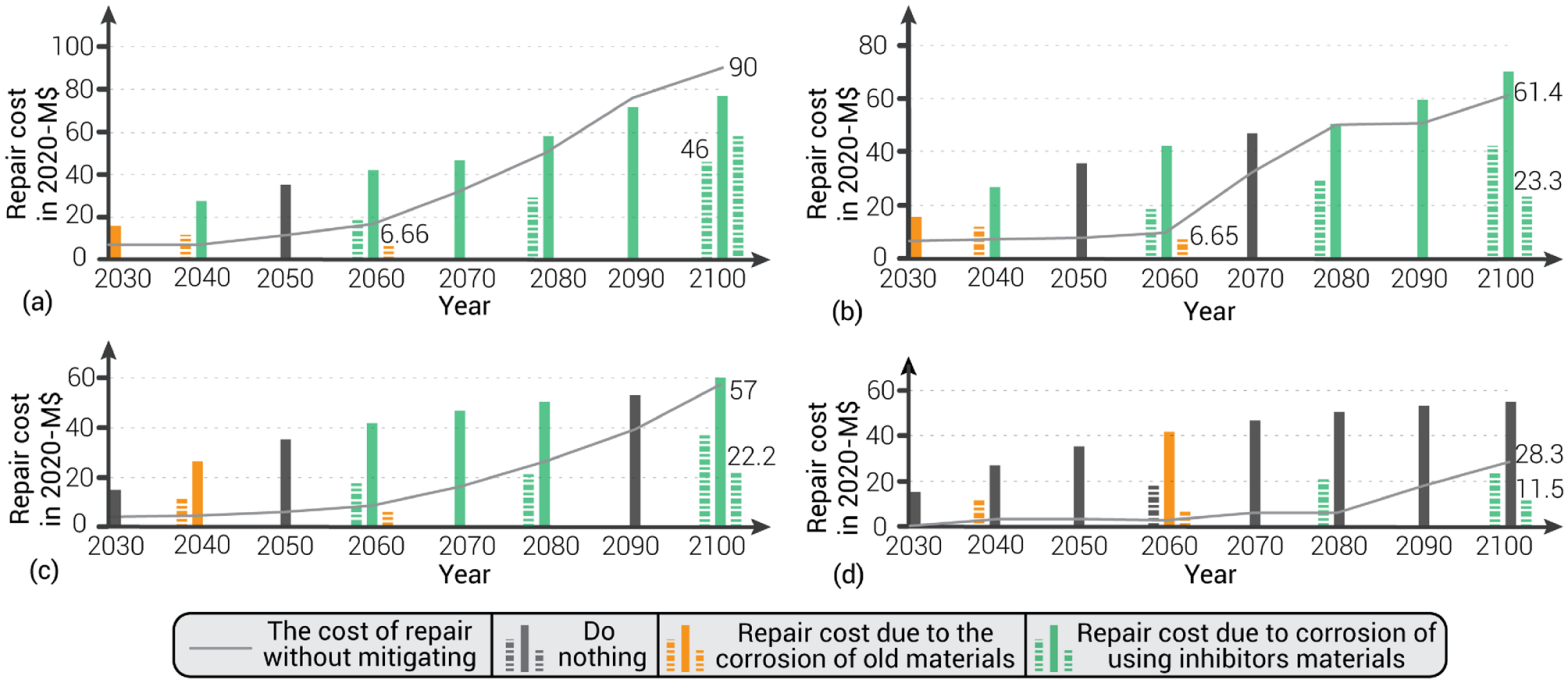
The total accumulated repair costs (in million 2020-$) using 10 years (middle column—option A), 20 years (left dashed column—option B), and 40 years (right dashed column – option C) periodic inspections under (**a**) SW1, (**b**) SW2, (**c**) SW3, and (**d**) SW4 scenarios.

While some buildings were inspected but not repaired, Fig. 5 shows if any buildings were repaired at any year. For example, some buildings were repaired under option A due to SW1 for all years except 2050. No buildings in any region were repaired in 2050 and 2060 under SW2 using strategy option A. Similarly, no buildings exposed to SW3 were repaired in 2030, 2050, and 2090 under option A. The relatively frequent inspection under option A resulted in the first repairs (corrosion due to old materials) for some buildings only in 2060 under SW4. For strategy option B, some buildings were repaired in all inspected years under all scenarios except the year 2060 under SW4. Some buildings were repaired for the first time in 2060 (corrosion in old materials) and for the second time in 2100 (corrosion in materials in which inhibitors may have been used) in strategy option C, regardless of which scenario was used.

For the life cycle analysis of all mitigation options, we used $325^46^ as the average home inspection cost for each building in vulnerable regions. This cost is added to the repair cost when the cracks appear on the concrete surface. Figure 5 shows the total costs for all buildings added up, discounted to present value, required to repair buildings’ foundation under each SWI scenario. The cost of the inspection in the year 2030 in vulnerable regions defined above will be around $15.2 million, as shown in Fig. 5c,d. The optimal strategy for minimizing the costs of periodic inspection and restoration at the community level under all SWI scenarios at the year 2060 is option C (every 40 years). In the year 2100, the combined cost of option C will be 38%, 39%, and 40% of the cost without mitigation under SW2, SW3, and SW4, respectively. Option B (every 20 years) is the optimal strategy under SW1 as it costs the community 51% of the cost of repair without mitigation in the year 2100 (Fig. 5a). The present combined cost of option B is lower than option A and option C by 40% and 20%, respectively, at the year 2100, under SW1.

The most cost-effective option for individual homeowners under SW1 is to inspect and repair their homes every 10 years (option A). In 2100, option A costs 30% and 60% less than options B and C, respectively. Option B costs 27% less than option A in 2040, but on the contrary, option A costs 24% less than option B in 2080. Supplementary Information S3 and Supplementary Fig. S3.3 explain the detailed analysis of the optimal inspection cost at the individual homeowner level.

## Discussion

Assessment of the potential impacts of four plausible SWI scenarios revealed that building foundations may be vulnerable to corrosion even under the mildest scenario (SW4). While high exposure of the foundations to chlorides under scenario SW1 had only a minor impact on residential buildings (around 2% by 2030), 43% of building foundations suffered from different levels of deterioration (up to 25% corrosion) by 2100. Most vulnerable buildings were located near the shoreline, as anticipated. However, buildings further inland were not impervious to corrosion, especially under the most severe scenario (SW1). If the foundations were found to be corroded in 2080 under scenario SW1, the total repair cost would be $50.35 million (in PV). A delay in inspection would lead to almost double the repair costs in 2080 under SW1 by 2100. These costs do not account for any direct/indirect effect of foundation damage on other structural damages of the buildings. Temporary relocation of residents of impacted buildings adds an estimated $2.39 million (in PV) to the cost of repairing corroded foundations under SW1 in year 2100. Since the region studied is 3–10 m above sea level, these outcomes may be amplified in low-lying regions (such as Florida or Louisiana) or coastal communities more susceptible to high levels of SWI.

Expansive soils cause an estimated $2.3 billion in buildings damages^47^ and around $15 billion in buildings and infrastructure damages^48^ yearly in the US on a nation-wide basis. Our analysis indicates a damage cost to building foundations due to SWI driven by SLR of $90 million (in PV) in one coastal county with approximately 400,000 inhabitants by 2100. Using this number to approximate damage costs to 16 million buildings from Fig. 2a, assuming the same ground water table and SWI, yields approximately $7 billion (in PV), approximately three times the damage cost to buildings due to expansive soils in the US.

Additional data would lower the uncertainties in the analysis and would give a more accurate estimate of the impact of GWD on the foundations. The corrosion level exposed to each building can be better identified by obtaining data on the foundation level of each building and analyzing Mobile County's salinity concentration. In addition, more data is needed to prove the assumption that the probability of corrosion initiation correlates with the degree of damage. The results of this analysis can help decision-makers or residents of coastal communities to think about climate change from another perspective and how it might impact a building foundation, which is one of its most critical elements. Early inspections are essential to repair the foundation before damage propagates and leads to costly building damage and repairs. In addition, the building code might consider increasing the minimum required concrete cover for reinforcement or adding an impervious layer to protect the reinforcement in the foundation from exposure to saltwater intrusion.

The effectiveness of alternative mitigation approaches should be examined using a life cycle cost analysis. A study of three example mitigation methods involving different inspection protocols revealed that investing in mitigation depends on the decision maker's perspective—individual homeowner vs, e.g., city building department. Inspection every 20 years (option B) is the most cost-effective method for periodic inspection and restoration in community level under SW1 scenario for the year 2100. Option B minimizes the community expenditures by around 50% comparing to repairs without mitigation. However, on the individual buildings level, an inspection every 10 years (option A) is the optimum mitigation in the year 2100 under SW1.

## Methods

### Ground water depths in Mobile County, AL

Ground water depths (GWD) in wells across Alabama are provided by the Geological Survey of Alabama (GSA)^49^. We utilize this information to determine the GWD, measured with respect to ground level, at each of the 4,719 wells located in Mobile County (Supplementary Fig. S2.3a). The GWD measures were taken in different years; the oldest measure was in 1900, and the newest one was in 2017. The GWD from 1900 to 2000 has a mean measure of 48 m with a standard deviation (SD) of 30 m, while the mean decreased to 44 m and the SD increased to 34 m after 2000. Population growth and economic development in Mobile County are two reasons^50^ for the decrease in groundwater depth in recent years and, consequently, the increase in susceptibility of reinforced concrete foundations to SWI.

As shown in Supplementary Fig. S2.3a, the spatial distribution of the wells does not give good coverage of the area of interest. Thus, the wells can only provide GWD information for some of the buildings. Accordingly, the spatial distribution of the GWD over Mobile County was determined from the GWD data by a Kriging method^51^. The accuracy of the interpolated GWD was measured by the Root Mean Square Error (RMSE), which was approximately 7 m and was considered relatively high for the purpose of this study. Dividing the area of interest into 8 regions, as shown in Supplementary Fig. S2.3a, reduced the RMSE error to roughly 3 m in each of the regions; however, that still was judged to be unacceptably high. Thus, we clustered the wells dataset inside each of the eight regions (Supplementary Fig. S2.3a) to calculate the cumulative distribution function (CDF) of GWD for the regions (Supplementary Fig. S2.3b). The CDF of the GWD in the 4719 wells was assumed to be lognormally distributed, using the following equation:

1 $$\text{P}\left(GWL \le {x}_{1}\right) =\Phi \left(\frac{(ln {x}_{1}) - \theta }{\omega }\right)$$

2 $${\omega }^{2}=\text{ln }(1 + \frac{\sigma }{\mu })$$

3 $$\theta =\text{ln }(\mu )- \frac{1}{2}{\omega }^{2}$$

where *Φ( )* = standard normal CDF, *x*_*1*_ is the state variable, $\mu$ and $\sigma$ are the mean and the standard deviation of the GWD in each region, and (*θ, ω*^*2*^) = the logarithmic mean and logarithmic variance of the lognormal distribution.

Equation (1) is used to estimate the probability of a particular GWD value in the year 2020 for each region. For the GWD decrease, we assume that the saltwater under the freshwater level is closely linked to ocean fluctuations^52^, as noted previously, and hence the GWD is assumed to be linearly related to SLR^12,13^. In addition, based on the slow increase in sea level through the years and assuming the GWD in wells data has reached a stationary condition, the variations in water permeability (hydraulic conductivity usually ranges 1–50 cm/h^53^) for different soil type (Supplementary Fig. S2.1) are not considered in this study. Supplementary Fig. S2.3c shows the anticipated SLR scenarios based on NOAA projections^54^ which has been used in previous studies^6,8^. The ground water estimation is summarized in step 2(b) in Fig. 3.

### Estimated foundation depth (FD)

Two sources of data were used to identify soil types, as shown in Supplementary Fig. S2.1. The first is the Soil Survey Geographic Database (SSURGO)^55^, which covers 75% of the area. The second is STATSGO, which gives generic (lower resolution) soil maps by state^55^.

We used the following equation to determine the FD’s range based on the soil type at the site of each building^56^:

4 $${D}_{min}= {\frac{q}{\gamma } \left[\frac{1 - sin \; {\upvarphi }}{1 + sin \; {\upvarphi }}\right]}^{2}$$

where D_min_ is the minimum depth of the foundation (the FD), ${\upvarphi }$ is the angle of repose (angle of friction), q is the bearing capacity of the soil, and $\gamma$ is the density of the soil. Supplementary Table S2.1 gives more details for the values of each variable used in Eq. (4).

### Corrosion of steel in concrete foundations

Corrosion of reinforcement in concrete foundations is most often caused by external sources of chlorides, including deicing agents and saltwater, as well as concrete ingredients. When steel is embedded in concrete, it is naturally protected (passivated) against corrosion because of the high pH (alkaline) environment of the cement. Chloride ions penetrate the concrete cover through capillary absorption, hydrostatic pressure, and diffusion^57^. Fick's second law models the diffusion mechanism of chloride penetration^22,58^.

5 $$C({x}_{2},t) = {C}_{0}\left[1-erf(\frac{{x}_{2}}{2\sqrt{Dt}})\right]$$

where erf ( ) is the error function, D is the diffusion coefficient, C_0_ is the surface chloride content, and C(x_2_,t) is the chloride content at a distance x_2_ in meters from the concrete surface at time t in years. Depassivation of the reinforcing steel occurs when chloride ions penetrate the concrete cover to the level of the reinforcement. Corrosion initiates when the chloride concentration at the reinforcing steel exceeds a threshold level, C_th_. Supplementary Fig. S2.5 shows the probability of corrosion initiation (P(CI)) corresponding to each SWI scenario in Table 1 by modifying Eq. (5) to determine the corrosion initiation time t, when C(x_2_, t) is replaced with C_th_. The mean and standard deviation of corrosion initiation are predicted using Monte Carlo Simulation (MCS). Supplementary Table S2.2 gives more details of the random variables used in MCS and Eq. (5). The P(CI) is estimated as P(GWD ≤ x_1_) $\cap$ P(CI ≤ x_2_) = P(CI ≤ x_2_|GWD ≤ x_1_)*P(GWD ≤ x_1_). Stewart et al.^59^ concluded that in the case of a high corrosion rate, the probability of corrosion damage is only slightly less than the probability of corrosion initiation. Thus, this study considers a corrosion rate due to the high chloride level assumed in SWI scenarios and the probability of corrosion initiation is regarded as the same as the probability of corrosion damage. Subsequent deterioration in strength is determined by loss of reinforcement area, cracking and spalling due to the expansive products of corrosion^60^.

### Life cycle cost analysis

Life cycle costs associated with various corrosion damage mitigation strategies were calculated by using the following equation^61^:

6 $$FR =PR{\left(1+i\right)}^{N}$$

where FR is the future repair cost, PR is the present repair cost, i is the periodic inflation rate, and N is number of years. In this study, we used PR equal to $15,000 if 5% ≤ P(CI) < 20% based on findings from the HomeAdvisor website for the maximum repair cost of buildings' foundations^62^. In addition, a foundation replacement of $75,000^63^ is assumed if corrosion has reduced the steel cross-section to less than 80% of its original diameter^64^. Thus, we assumed to use PR equal to $75,000 if P(CI) ≥ 20%. For 1% ≤ P(CI) < 5% we assumed PR equal to $3000. The annual inflation rate was assumed to be 3%. The future repair cost is then multiplied by the probability of corrosion initiation to calculate the repair cost for each building at each year. Future costs of repairing, inspecting, and maintaining were computed using today's pricing and approximated using Eq. (6) and a forecast inflation rate for comparisons of mitigation strategies. Then, using Eq. (7) and a discount rate, the present values of future costs were estimated.

7 $$DPR = \frac{FR}{{(1+d)}^{N}}$$

where DPR is the discounted present repair cost, d is the nominal periodic discount rate. According to the United States Office of Management and Budget (OMB)^65^, the regulatory analysis real discount rate should be 7% or 3%, representing average private capital return in the US economy or the social rate of time preference, respectively. In this study we used the 6% discount rate to calculate the DPR value.

## Supplementary Information

Supplementary Information.

## Data Availability

The datasets used and/or analyzed during the current study available from the corresponding author on reasonable request.
