# Supplementary material for: Hidden costs to building foundations due to sea level rise in a changing climate
Source: Sci Rep. 2022 Aug 18;12:14020. doi: 10.1038/s41598-022-18467-3 (PMC9388630; doi:10.1038/s41598-022-18467-3)
Supplement: Supplementary file 1 — Supplementary Information. [file 41598_2022_18467_MOESM1_ESM.pdf]

SUPPLEMENTARY INFORMATION

Hidden costs to building foundations due to sea level rise in a changing climate

Mohamed A. Abdelhafez, Bruce Ellingwood, Hussam Mahmoud
Department of Civil and Environmental Engineering, Colorado State University, Fort Collins, CO, United States, Hussam.Mahmoud@colostate.edu

S1 Datasets for modeling building inventories and groundwater table in Mobile County, AL.

Microsoft data

Data related to the building inventory in Mobile County, AL, were obtained from the Microsoft Footprint database for the U.S. (Bing Maps dataset), which is the latest and most detailed building footprint database [1]. The Microsoft Bing Maps team published computer-generated building footprints from Bing satellite imagery in the United States in 2018 using the open-source Microsoft Cognitive Toolkit (CNTK). Five million annotated pictures were used to train the output of building footprints, and the results of buildings match reached a 98.5% accuracy with 2.5 degrees of rotation error as well as a 1% false-positive ratio in 1000 randomly selected structures from the whole output dataset [1]. Bing Maps provides building location and area by utilizing the deep learning methods for object classification in the U.S. (130 million buildings), Canada (12.6 million buildings), Uganda (7 million buildings) , and Tanzania (11 million buildings) [1]. The Microsoft building footprints are available for each state of the U.S. in GeoJSON format. A Python (3.8.5) code was written to convert the Alabama building dataset from GeoJSON to a Shapefile format using GeoPandas package (0.8.2) [2] for subsequent analysis using Geographic Information System (GIS) software such as QGIS 3.16 [3]. For accurate extraction of building dimensions and areas, we projected the Microsoft buildings footprint layer onto a Universal Transverse Mercator (UTM) Zone 16 - NAD83 projection. The final shapefile for building footprints in Mobile County consists of 176,516 polygons.

ATTOM data

ATTOM data [4] is a real estate information database, which is derived from public records. It includes approximately 300 property details such as coordinates (longitude and latitude), address (street, city, and state), cost (i.e., taxes), and size of buildings. Not all the 300 attributes were available for each building. For this paper, we used the values in the field “property17” of ATTOM data to find the type of buildings. A Residential building is coded “385”, a commercial building is coded “165”, other buildings are coded “379”, and an unknown building is coded “0”. Number of stories for residential buildings was identified by using the values in the filed "storiescou". The values in the filed “yearbuilteffective” were used to identify the age of the residential buildings.

One of the critical attributes required for matching the ATTOM data with the Microsoft footprint data is the building coordinates information. Thus, we used Python code to find the missing coordinates data for those buildings with address details (i.e., house number, street name, zip code, etc.) using the Geocoding services. Web search engines are the most common source of geocoding information and tools. The application programming interface (API) offered by some web search engines, such as google maps, allows developers or researchers to create user-specific codes using their APIs. We geocoded the missing coordinates using the Google Maps API [5] through the GeoPy python module [6]. The geocoding returns the results in the WGS84 projection, which were reprojected to (UTM) Zone 16 - NAD83 to be consistent with Microsoft Buildings' footprint.

Merging Microsoft and ATTOM data

The merging process between Microsoft buildings' footprint (176,516 polygons) and ATTOM data (115,490 points) was done in two steps due to a lack of enough points from the ATTOM data to match with all buildings in Microsoft footprint. Furthermore, some of ATTOM points represent more than one building in case if those buildings have the same type of information. Thus, most of the ATTOM data points are not located inside the corresponding Microsoft buildings.

If two items in separate data sets fulfill the spatial operator "Intersect," "Overlap," "Contains," or "within," then the spatial join tool in QGIS is a helpful tool to be used. Thus, the attribute information from the point shapefile (ATTOM) was copied to the target polygons of the building outlines in Microsoft shapefile using the spatial join tool of QGIS. Then, the first step of the matching process between the two datasets was done using the intersect operator from the spatial join tool of QGIS. This step enabled the attributes from the ATTOM data to be merged to approximately 50% of the Microsoft polygons. In the second step, we applied the "join attributes by nearest" function of QGIS on the rest of the unmatched buildings in the Microsoft shapefile. This function gives more control than the "spatial join" function as it allows to specify the maximum distance between the input layer (ATTOM's points) and the joined layer (Microsoft's polygons). Using 60 meters as the maximum distance in the merging process between the two datasets yielded a 96% match (169,906 buildings). The remaining 4% of the unmatched Microsoft's buildings were ignored as there were no data points from the ATTOM data that were 60 meters away. Most of those buildings were not identified using Google Maps as residential buildings.

Ground water data

The Geological Survey of Alabama (GSA) provides data on locations and ground water depths (GWD) in wells in all counties in Alabama [7]. These data were stored in ArcGIS MapServers, an Esri [8] product to manage and disseminate GIS data. We used the esri2geojson command line to fetch the wells data from the Mapservers. Then we used a Python (3.8.5) code to convert the wells dataset from GeoJSON to Shapefile format using GeoPandas package (0.8.2) [2]. The resulting shapefile gives 15 attributes for 264,762 wells in Alabama. The attributes we used in this study were “MeasurementDate” and “WaterLevel”. The GWD information was extracted from “WaterLevel” values, which are measured from the ground level at the measurement date. The values inside “MeasurementDate” were in int64 format; thus, we used the datetime package [9] in

Python to convert it to Isoformat to extract the measured year. The “Extract by location” function of QGIS was used to find the 4,719 wells inside Mobile County and store them in a separate shapefile.

S2. Supporting figures and tables for modeling the corrosion of steel in concrete foundations

Figure S2.1 depicts the two data sources utilized to classify soil types. SSURGO, which covers a portion of the region. In addition, STATSGO provides general (lower resolution) maps of soils by country [10].The soil types data were used in the FD calculations as explained in the Methods. Figure S2.2 illustrates that the elevations of Mobile and Baldwin Counties are sufficiently comparable to be utilized as assumptions for SWI scenarios. Figure S2.3 (b) shows that the CDF of GWD for Mobile County has been partitioned from the divided eight regions from Figure S2.3 (a). The relative SLR projected at NOAA’s station in Dauphin Island, AL [11], is used to represent the future SLR in this study, as seen in Figure S2.3 (c). Since the structural behavior of building foundations affects occupant safety, and since the GWD in the area of interest (Figure S2.3 (a)) is relatively high, we selected the extreme relative SLR scenario (on NOAA's scale [12]), which led to 3.28 m in the year 2100 (Figure S2.3 (c)). This correspond to about 0.1% probability of exceedance for RCP 8.5 in IPCC’s scale [13]. NOAA's forecasts are conservative [14], yet SLR in the Gulf of Mexico will be higher than the global average for all RCPs [15]. Figures S2.4 and S2.5 show the corrosion initiation probabilities, with and without employing the inhibitors materials indicated in Methods section. The FD's minimum and maximum values for each soil type are determined according to Table S2.1 and the Methods section. The number of buildings with their number of floors for each soil type is shown in Table S2.3.

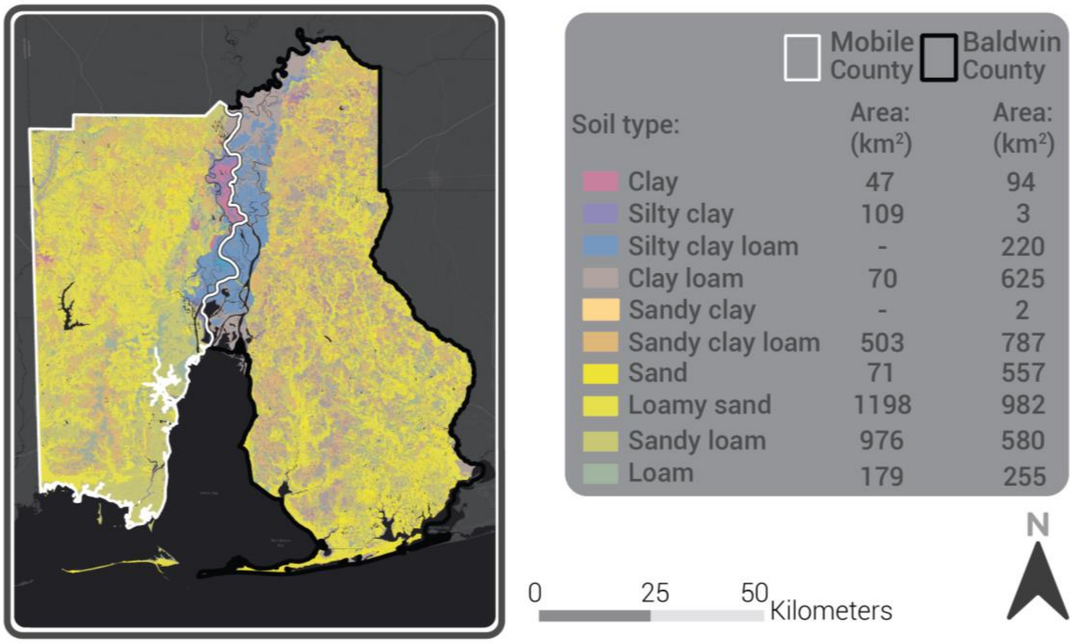

Supplementary Figure S2.1: Soil types in Mobile and Baldwin counties [10]. **The software used to develop the map in this figure is QGIS 3.16 (https://qgis.org).**

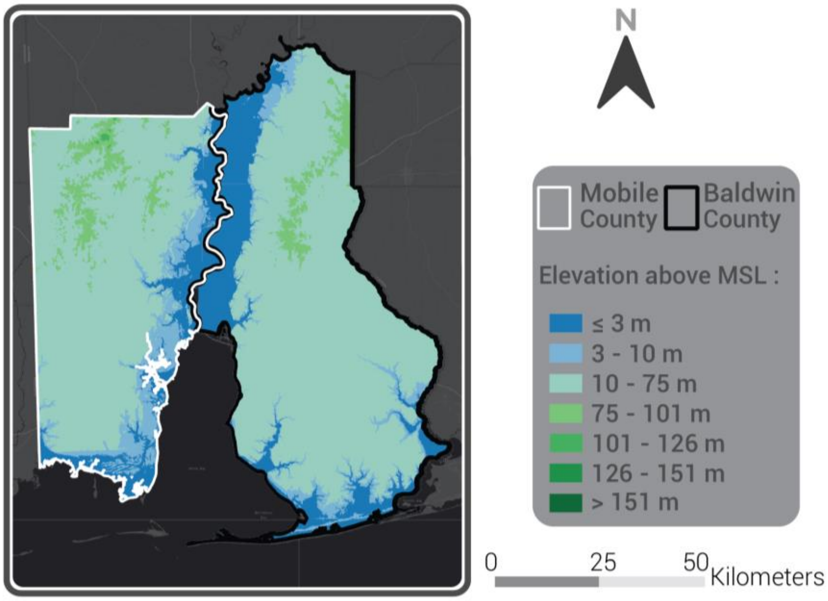

Supplementary Figure S2.2: Digital elevation model (DEM) for Mobile County and Baldwin County [16]**. The software used to develop the map in this figure is QGIS 3.16 (https://qgis.org).**

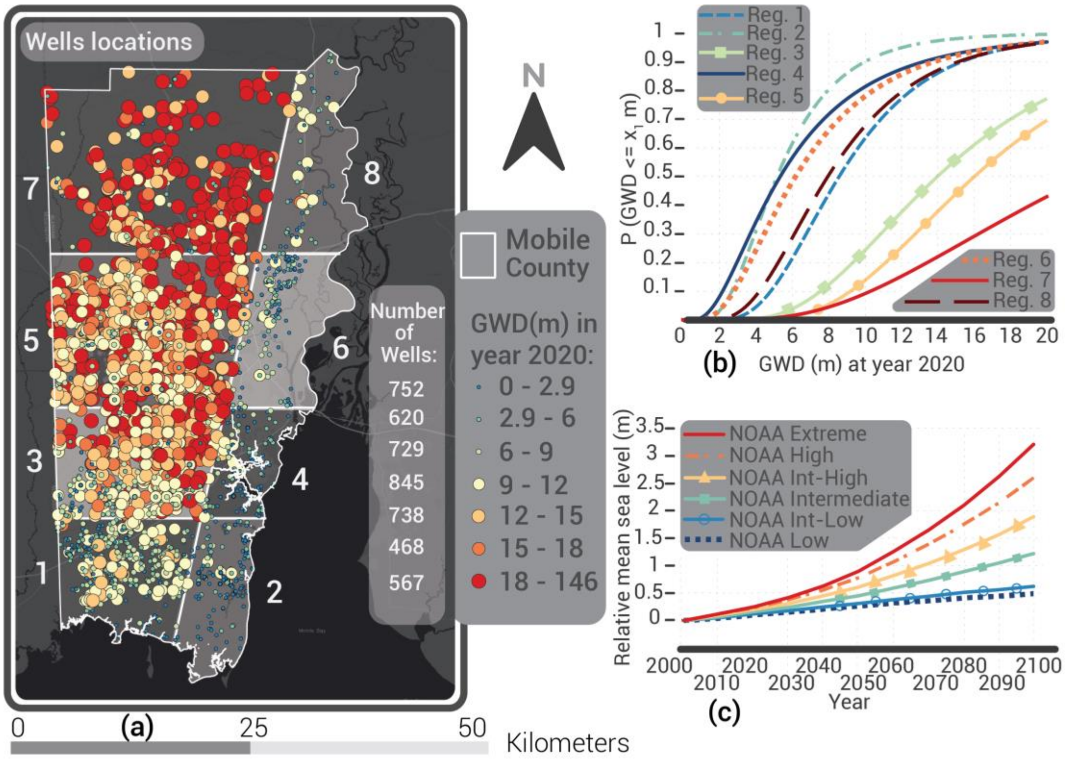

Supplementary Figure S2.3: (a) Number of Wells with their GWD measured from ground level, (b) the CDF of the clustered regions, and (c) NOAA's projections for relative SLR in Dauphin Island. The software used to develop the map in this figure is QGIS 3.16 (https://qgis.org).

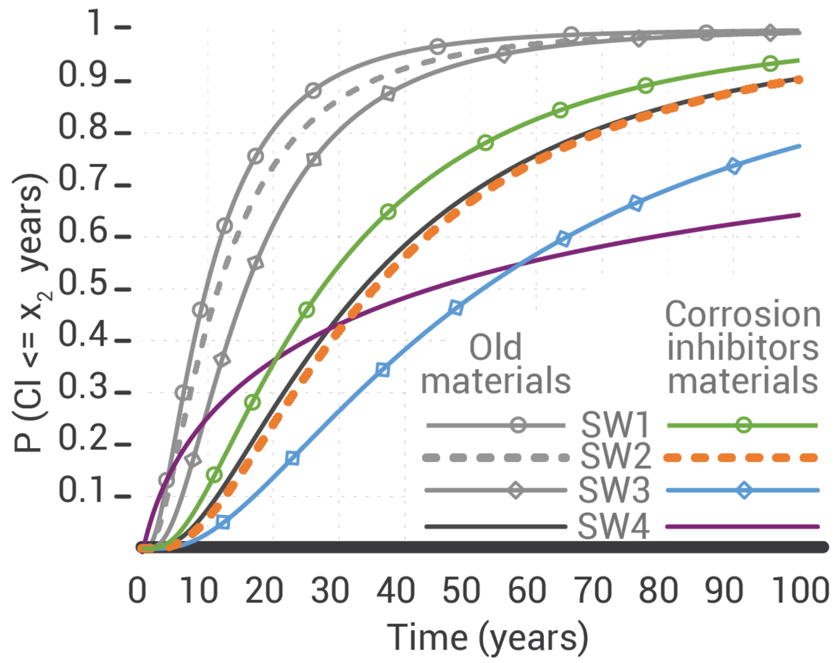

Supplementary Figure S2.4: Probability of corrosion initiation due to SWI scenarios using corrosion inhibitors materials.

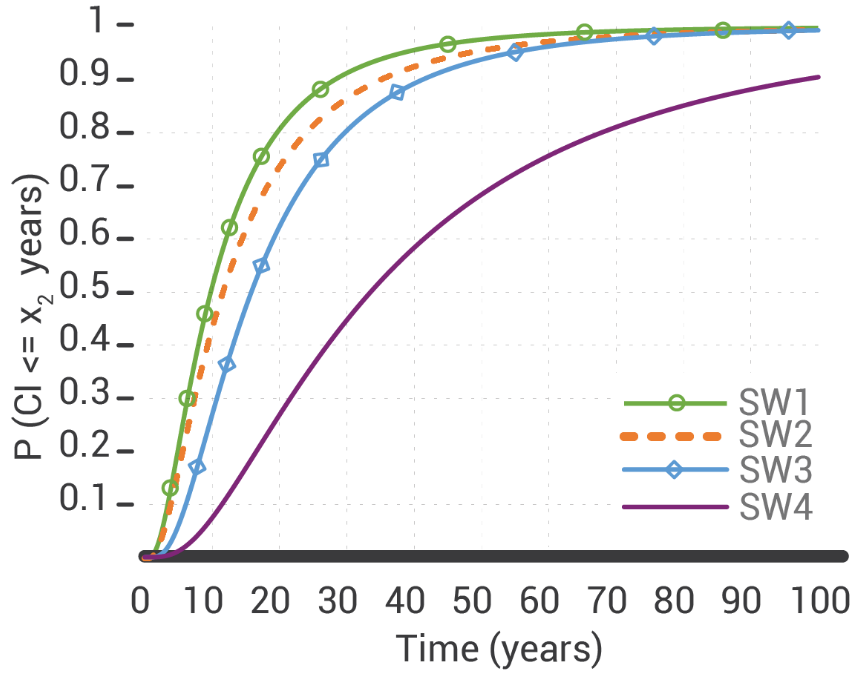

Supplementary Figure S2.5: Probability of corrosion initiation due to SWI scenarios.

Supplementary Table S2.1: Statistical parameters of the variables used to estimate the FD for each soil type

Soil type	Soil Texture [17]	Angle of Repose [Reference]	Bearing capacity (KN/m^2) [Reference]	Density (KN/m^3) [Reference]	Minimum Depth, FD (m)
Clay	40% < clay < 100%<br>40% < silt < 100%<br>0% < sand < 45%	18-28 [18]	215-270 [19]	13 [20]	2.15 - 5.78
Silty clay	40% < clay < 60%<br>40% < silt < 60%<br>0% < sand < 20%	18-32 [18]	200 [21]	12.41 [20]	1.52 - 4.49
Silty clay loam	27.5% < clay < 40%<br>40% < silt < 72.5%<br>0% < sand < 20%	18-32 [18], [22]	200 [23]	12.72 [20]	1.48 - 4.38
Clay loam	27.5% < clay < 40%<br>15% < silt < 52.5%<br>20% < sand < 45%	18-32 [18]	200 [23]	13.66 [20]	1.38 - 4.07
Sandy clay	35% < clay < 55%<br>0% < silt < 20%<br>45% < sand < 65%	27-35 [18]	200 [23]	14.45 [20]	1.01 - 1.95
Sandy clay loam	20% < clay < 35%<br>0% < silt < 27.5%<br>45% < sand < 80%	31-34 [18]	215-270 [19]	14.75 [20]	1.16 - 1.88
Sand	0% < clay < 10%<br>0% < silt < 15%<br>85% < sand < 100%	37-38 [18]	100-300 [24]	14 [20]	0.4 - 1.32
Loamy sand	0% < clay < 15%<br>0% < silt < 30%<br>70% < sand < 85%	32-35 [18]	80-160 [25]	14 [20]	1.0 - 1.39
Sandy loam	0% < clay < 20%<br>0% < silt < 50%<br>42.5% < sand < 70%	27-35 [18]	80-160 [19]	14.3 [20]	0.44 - 1.58
Loam	7.5% < clay < 27.5%<br>27.5% < silt < 50%<br>22.5% < sand < 52.5%	28-32 [18]	80-160 [19]	14 [20]	0.54 - 1.49

Supplementary Table S2.2: Statistical parameters of random variables used in MCS of probability of corrosion initiation

Parameter	Mean	COV	Distribution	Reference
x_2 (mm)	60	0.2	Lognormal	[26]
D (mm^2/year)	63	0.75	Lognormal	[26]
C_{th} (kg/m^3)	1	0.1	Lognormal	[27]
C_0 (kg/m^3) (ppt)	Table 1	0.1	Lognormal	

Supplementary Table S2.3: Number of buildings for each soil class in each region

Soil type	Region 1		Region 2		Region 3		Region 4		Region 5		Region 6		Region 7		Region 8	
	# of stories		# of stories		# of stories		# of stories		# of stories		# of stories		# of stories		# of stories	
	1	≥ 2	1	≥ 2	1	≥ 2	1	≥ 2	1	≥ 2	1	≥ 2	1	≥ 2	1	≥ 2
Clay	0	0	0	0	0	51	0	0	51	1	0	0	1	0	72	3
Silty clay	0	0	0	0	0	0	4	0	0	0	0	0	0	0	0	0
Silty clay loam	0	0	0	0	0	0	0	0	0	0	0	1	0	0	0	0
Clay loam	31	1	3	0	56	2	0	0	6	1	71	4	12	2	31	2
Sandy clay	0	0	0	0	0	0	0	0	0	0	0	0	0	0	0	0
Sandy clay loam	2594	104	57	4	6092	700	0	0	2802	141	696	22	1141	105	558	23
Sand	0	0	1	0	0	0	0	0	30	3	230	17	203	4	26	6
Loamy sand	1618	53	283	5	15373	4023	3084	405	15087	1759	1342	423	760	40	89	1
Sandy loam	3344	95	2016	121	8739	1292	20601	1983	6375	299	22745	1147	840	35	487	19
Loam	760	33	50	4	2066	210	491	646	372	18	860	917	0	0	10	1

S3. Foundation corrosion and mitigation strategies.

Figures S3.1 and S3.2 show the spatial distribution of the residential buildings with their levels of foundation corrosion due to SW2, SW3, and SW4. In comparison with SW1, SW2 caused only a minor probability of corrosion initiation (1-5%) on roughly 2% of all residential buildings by the year 2030, as shown in Figure S3.1. By 2100, 17% and 6% of the building foundations had probabilities of corrosion equal to 5-10% due to SW2 (Figure S3.1) and SW4 (Figure S3.2), respectively, while 5-10% corrosion occurred due to SW3 in 18% of building foundations by the year 2100. Around 7% and 5.5% of buildings located in vulnerable regions had 10-40% corrosion under SW2 and SW3, respectively. The exposure scenarios SW4, SW3, and SW2 showed different levels of corrosion to 43%, 43%, 44% of the buildings by 2100. However, the number of buildings with high corrosion levels (15-40%) under SW2 is almost double that of buildings under SW3. As shown in Figure S3.2, only a few buildings had a 10-40% probability of corrosion initiation by the year 2100 under the lowest exposure scenario (SW4). Figure S3.2 indicates that even with the lowest exposure SW4, around 43% of the buildings located in regions 2, 4, and 6 will still be vulnerable to SWI by having a 1-10% chance of corrosion by 2100.

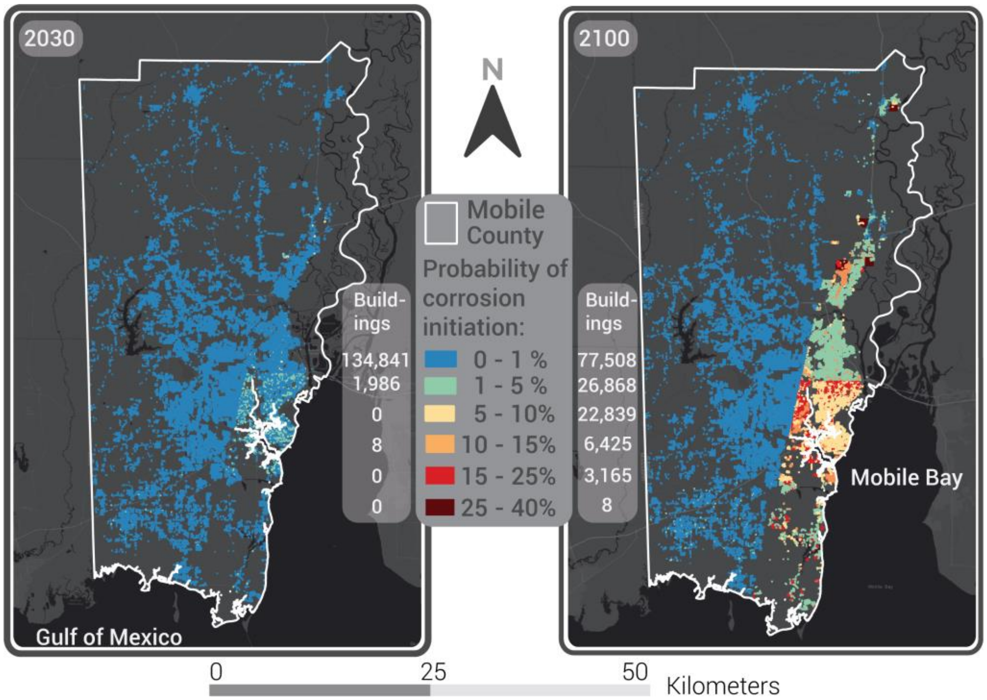

Supplementary Figure S3.1: Probability of corrosion initiation due to SW2 in years 2030 and 2100. The software used to develop the maps in this figure is QGIS 3.16 (https://qgis.org).

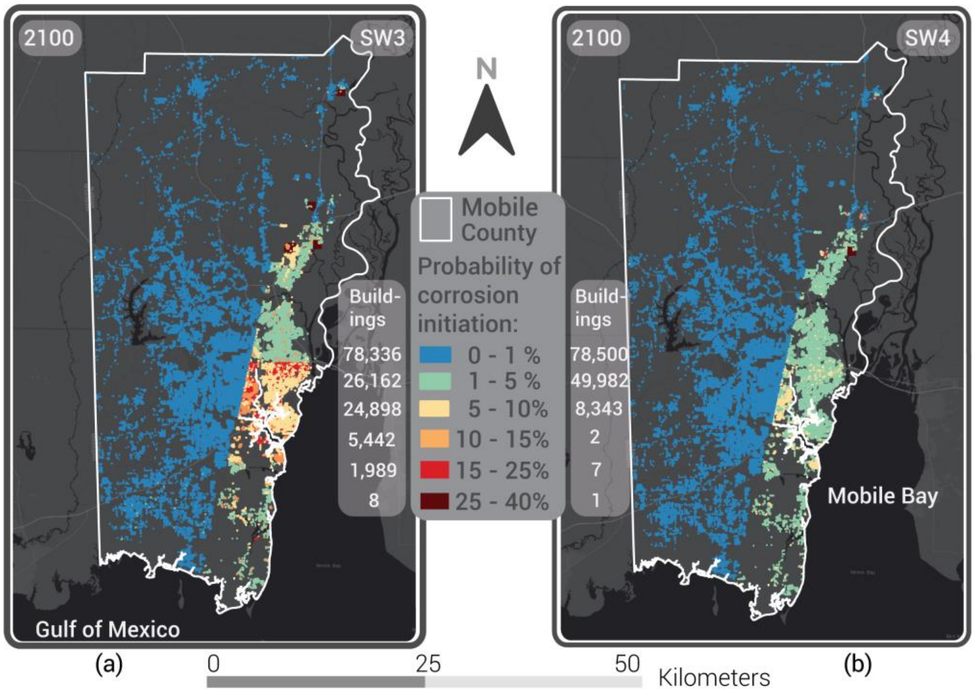

Supplementary Figure S3.2: Probability of corrosion initiation due to (a) SW3 and (b) SW4 in year 2100. The software used to develop the maps in this figure is QGIS 3.16 (https://qgis.org).

Figures S3.3 illustrate the life-cycle analysis of multi-story buildings located on clay loam soil in region 6 under the three mitigation strategies - Options A, B and C - considered previously. We assume that 10% corrosion is indicative of a hairline crack and is a threshold for repairing the building foundation. Assuming that the foundation has been exposed to chloride for 10 years starting in 2020, the inspection for option A (every ten years) (Figure S3.3 (a)) is expected to begin in 2030. Because corrosion is not permitted for more than ten years, we assume for this technique that only P1 (from (2-d) in figure 3) would be computed. In this example, the corrosion exceeded the threshold level in the year 2030, so the foundation was repaired and inhibitors were applied to resist corrosion in subsequent years. Thus, from the year 2040 to the year 2100, no hairline cracks were found, and no repairs were made. The inhibitor materials for this example, lowered the corrosion below the corrosion of doing nothing and below the threshold level.

Figure S3.3 (b) shows the life-cycle analysis for Option B (every 20 years inspection), with inspections beginning in 2040. For this option, both P1 (10 years exposure) and P2 (20 years exposure (from (2-d) in figure 3) are computed since corrosion is not permitted to increase for more than 20 years. In this case, corrosion exceeded the threshold level in 2040; thus the foundation is repaired and inhibitor materials are applied to combat corrosion in subsequent years. For the years 2060, 2080, and 2100, hairline cracks were detected in the foundations and repairs were made. Since the inspection is less frequent in Option B than in Option A, corrosion under Option B exceeded that under Option A and corrosion exceeded the threshold level, as shown in Figure S3.3 (b), even when inhibitor materials were used.

For option C (every 40 years inspection) (Figure S3.3 (c)), the inspection is considered to start in 2060. This option requires that P1 to P4 (figure 3) are calculated since corrosion cannot grow for more than 40 years. Hairline cracks in the foundations were detected in 2060 and 2100; foundations were repaired at those inspections and corrosion-inhibitors were used to prevent further corrosion. Option C has a greater probability of corrosion initiation than option B since inspections are performed less often. Figure S3.3 (c) shows that even with the use of inhibitor materials, corrosion surpassed the threshold level.

As mentioned before in the main paper, we assumed an average home inspection cost of $325 for each susceptible building in our life cycle analysis of all mitigating strategies. This cost is added to the repair cost when the cracks appear on the concrete surface. Figure S3.3 shows the total costs for a building in region 6 located on clay loam soil, discounted to present value, required to repair buildings' foundation under scenario SW1. Option A (inspection/repair every 10 years) is the most cost-effective option for reducing the expenses of periodic inspection and restoration on the individual building level under scenario SW1. The present combined cost of option A is lower than option B and option C by 30% and 60%, respectively, at the year 2100. Using a 20-year life span as a benchmark, the cost of option B under SW1 is 27% less than the cost of option A in 2040. However, option A is lower than option B by 24% in 2080 when the building owners compares the two alternatives.

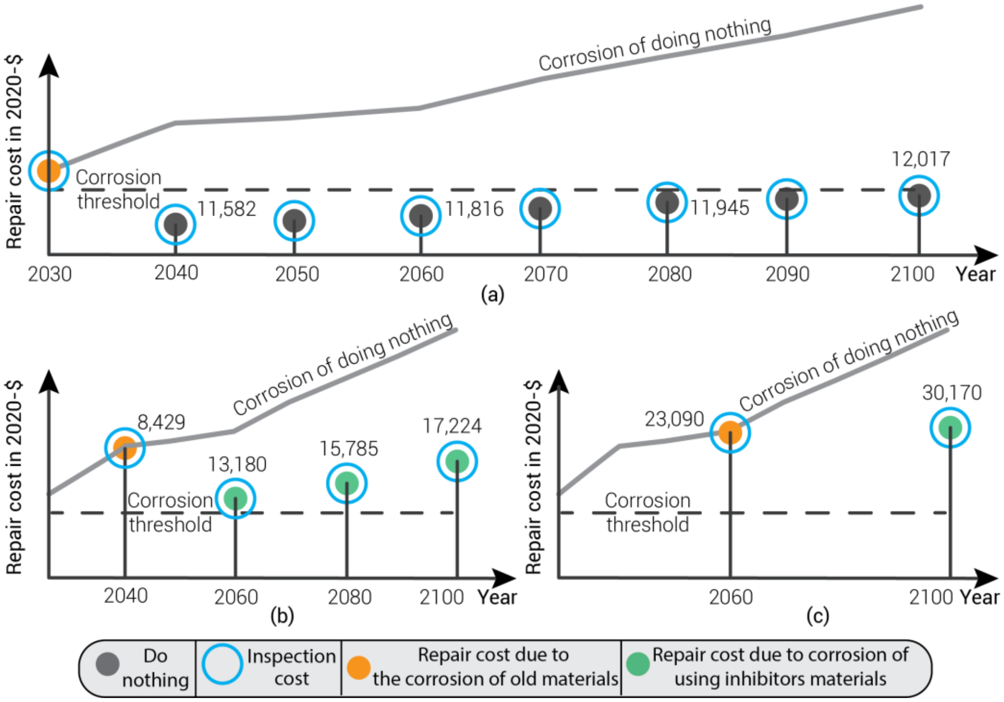

Supplementary Figure S3.3: The total accumulated repair costs (in 2020-$) using a) 10 years (option A), b) 20 years (option B), and c) 40 years (option C) periodic inspections for a building in region 6 on clay loam soil type with maximum FD under SW1.

Figures S3.4 - S3.7 show the probability of corrosion initiation (P(CI)) based on the concept illustrated in Figure S3.3 in two sample buildings erected on clay loam soil with minimum FD (one-story) and with maximum FD (multiple stories) using periodic inspection every 10 years (Option A) and 20 years (option B) mitigation techniques. To calculate the expected cost of repairs under periodic inspection and mitigation, the probability of corrosion initiation is calculated only if it exceeds the threshold level (10%); otherwise, it is set equal to zero. Hence, a one-story building located on clay loam soil would not need any repairs prior to 2100 under scenarios SW3, and SW4 and Option A, as shown in Figure S3.4 and Figure S3.6. Furthermore, only buildings located in regions 2 and 6 exceeded the threshold level by 2090 and 2100, respectively, under scenario SW1. Thus, repairs are conducted for those buildings in the years 2090 and 2100 using inhibitors materials. In the year 2100, the corrosion in buildings of region 2 did not exceed the threshold level because of the repairs conducted in the year 2090.

For multi-story buildings located on clay loam soil in region 8, no repair costs under scenarios SW1 and SW2 are incurred up to the years 2070 and 2080, respectively, at which time the threshold is exceeded (Figure S3.4). The corrosion inhibitors used in the repairs at 2070 and 2080 resisted corrosion in 2080 (for SW1) and 2090 (for SW2), so no repairs. Similarly, buildings in region 1 required repairs only in the year 2090 under SW1 and SW2. Buildings in region 6 need only repairs at the year 2030 under SW1 and SW2. No repairs are indicated for buildings in regions 2,3,4,5 and 7 under all scenarios. No cracks appeared in any building at any year under SW3 and SW4 (Figure S3.6).

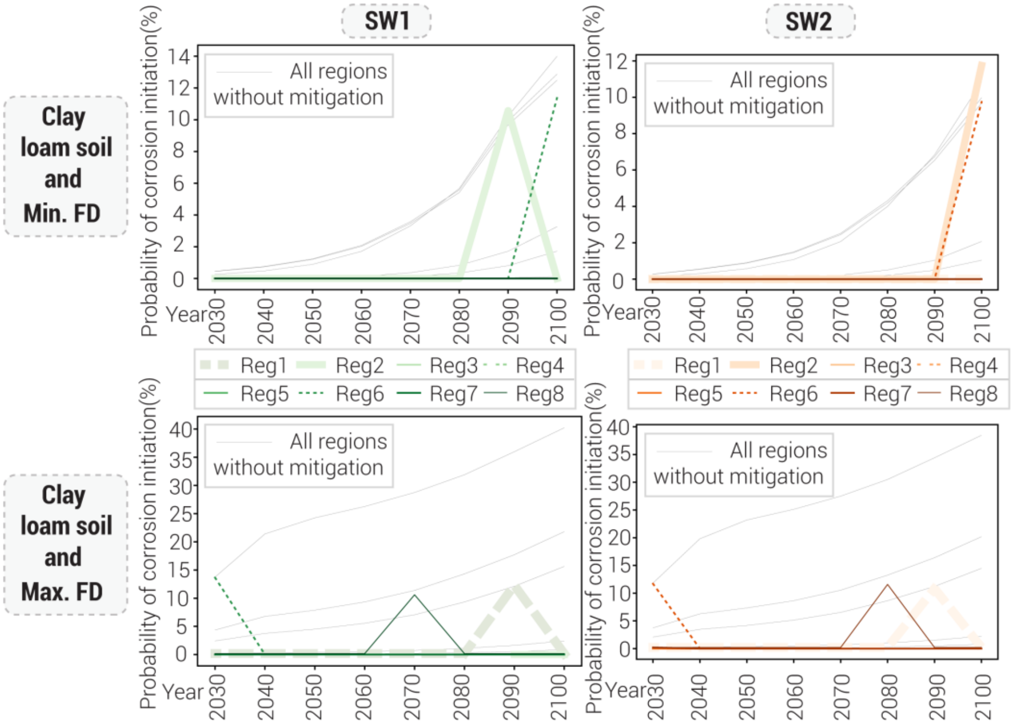

Supplementary Figure S3.4: Probability of corrosion initiation for a soil type under SW1 and SW2 using periodic inspection every 10 years (option A) mitigation strategy.

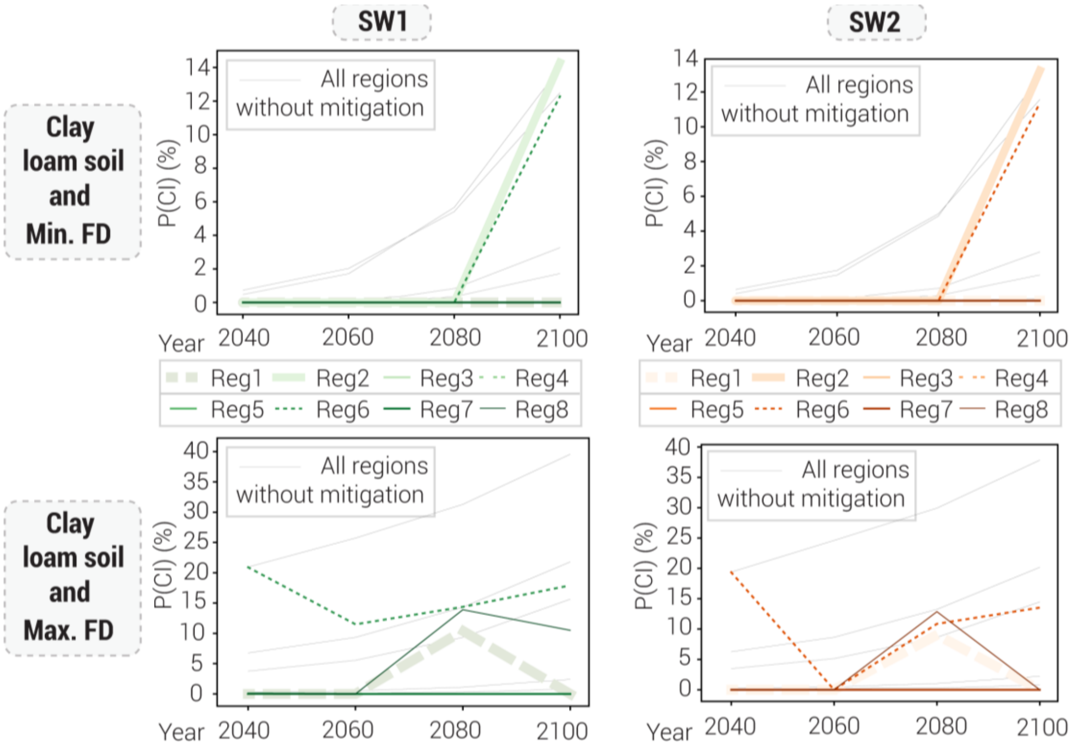

Supplementary Figure S3.5: Probability of corrosion initiation for a soil type under SW1 and SW2 using periodic inspection every 20 years (option B) mitigation strategy.

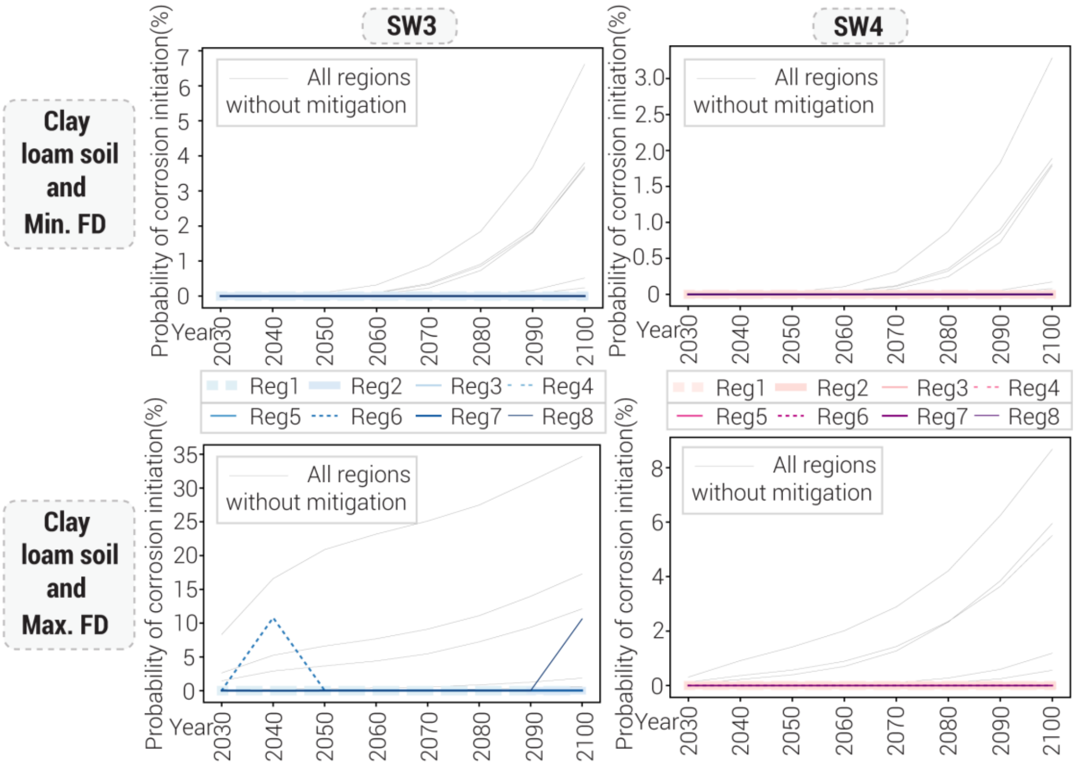

Supplementary Figure S3.6: Probability of corrosion initiation for a soil type under SW3 and SW4 using periodic inspection every 10 years (option A) mitigation strategy.

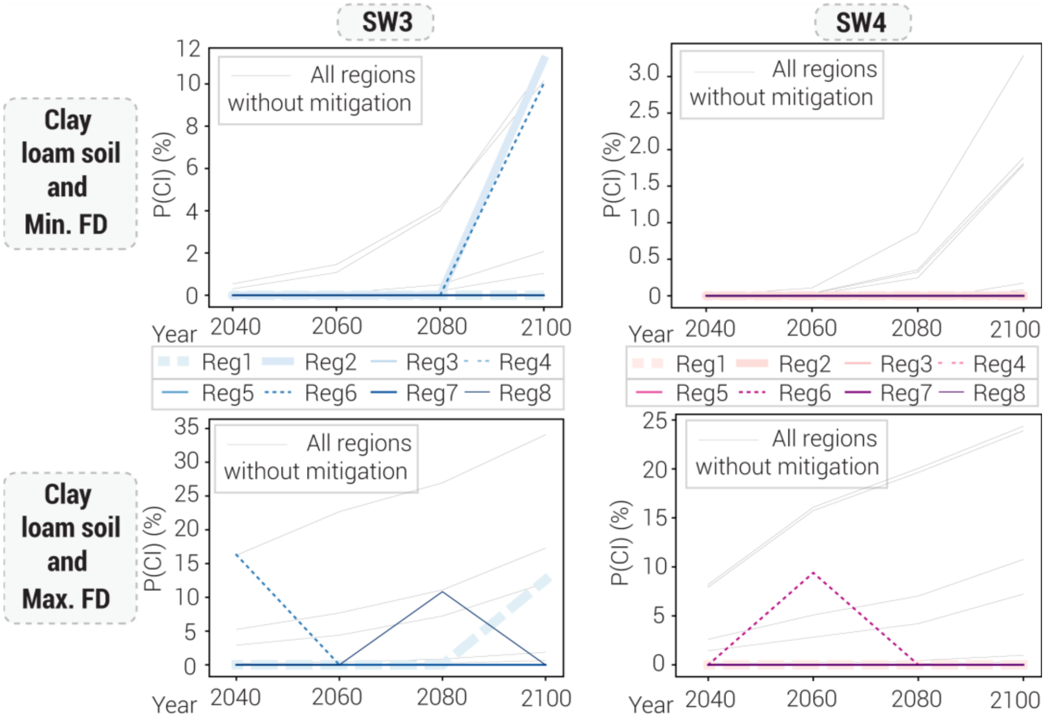

Supplementary Figure S3.7: Probability of corrosion initiation for a soil type under SW3 and SW4 using periodic inspection every 20 years (option B) mitigation strategy.

References

[1] Microsoft, “Bing Maps Team. Computer Generated Building Footprints for the United States.,” 2018. [Online]. Available: https://www.microsoft.com/en-us/maps/building-footprints.

[2] GeoPandas, “An open source project to make working with geospatial data in python easier.,” 2021. [Online]. Available: https://geopandas.org/. [Accessed: 10-Jun-2021].

[3] QGIS, “A Free and Open Source Geographic Information System - version 3.16,” 2021. [Online]. Available: https://qgis.org/en/site/. [Accessed: 10-Jun-2021].

[4] ATTOM, “ATTOM Data Solutions,” 2021. [Online]. Available: www.attomdata.com. [Accessed: 10-Jun-2021].

[5] Google Maps Platform, “Geocoding API,” 2021. [Online]. Available: https://developers.google.com/maps/documentation/geocoding/overview. [Accessed: 10-Jun-2021].

[6] Geopy, “A Python client for several popular geocoding web services.,” 2021. [Online]. Available: https://github.com/geopy/geopy. [Accessed: 10-Jun-2021].

[7] GSA, “Groundwater Assessment Program - Well Records,” 2021. [Online]. Available: https://www.gsa.state.al.us/gsa/groundwater/wellrecords. [Accessed: 22-Jun-2021].

[8] Esri, “The global market leader in GIS,” 2021. [Online]. Available: https://www.esri.com/en-us/home. [Accessed: 10-Jun-2021].

[9] datetime, “Basic date and time types.” [Online]. Available: https://docs.python.org/3/library/datetime.html. [Accessed: 10-Jun-2021].

[10] USGS, “Soil Survey Geographic Database (SSURGO) and Soil Geographic Database (STATSGO),” 2021. [Online]. Available: https://apps.nationalmap.gov/viewer/. [Accessed: 22-Jun-2021].

[11] NOAA, “Global and Regional Sea Level Rise Scenarios for the United States,” *NOAA Tech. Rep. NOS CO-OPS 083*, no. September, pp. 1–56, 2022.

[12] W. V. Sweet *et al.*, “Global and regional sea-level rise scenarios for the United States.,” *NOAA Tech. Rep. NOS CO-OPS 083*, no. January, 2017.

[13] IPCC, “Climate Change 2014: Synthesis Report. Contribution of Working Groups I, II and III to the Fifth Assessment Report of the Intergovernmental Panel on Climate Change . IPCC, Geneva, Switzerland, 151 pp. In IPCC AR5 Synthesis Report website.,” 2014.

[14] M. Ghanbari, M. Arabi, J. Obeysekera, and W. Sweet, “A Coherent Statistical Model for Coastal Flood Frequency Analysis Under Nonstationary Sea Level Conditions,” *Earth’s Futur.*, 2019.

[15] NOAA, “Climate Change: Global Sea Level,” 2021. [Online]. Available: https://www.climate.gov/news-features/understanding-climate/climate-change-global-sea-level. [Accessed: 04-Sep-2021].

[16] USDA, “Geo Spatial Data,” 2021. [Online]. Available: https://datagateway.nrcs.usda.gov/GDGOrder.aspx. [Accessed: 22-Jun-2021].

[17] G. G. Pohlman, “Soil Science Society of America,” *Soil Sci. Soc. Am. J.*, vol. 4, no. C, 1940.

[18] Geotechdata.info, “Angle of Friction,” 2021. [Online]. Available: http://geotechdata.info/parameter/angle-of-friction.html. [Accessed: 22-Jun-2021].

[19] FAO, “Footings and foundations,” 2021. [Online]. Available: http://www.fao.org/3/s1250e/S1250E0h.htm. [Accessed: 22-Jun-2021].

[20] Structx, “Density Ranges for Different Soil Types,” 2021. [Online]. Available: https://structx.com/Soil_Properties_002.html. [Accessed: 22-Jun-2021].

[21] P. K. Kolay, S. Kumar, and D. Tiwari, “Improvement of Bearing Capacity of Shallow Foundation on Geogrid Reinforced Silty Clay and Sand,” *J. Constr. Eng.*, vol. 2013, p. 293809, 2013.

[22] L. C. Van Rijn, “CHANNEL SLOPES OF MUD, SILT AND SAND.” [Online]. Available: https://www.leovanrijn-sediment.com/papers/Channelslopesbrief2018.pdf. [Accessed: 22-Jun-2021].

[23] C. of A. B. Officials, *CABO One & Two Family Dwelling Code*. Council of American Building Officials, 1983.

[24] Geotechdata.info, “Soil bearing capacity.” [Online]. Available: http://www.geotechdata.info/parameter/bearing-capacity. [Accessed: 22-Jun-2021].

[25] C. J. Hayes, “Bearing Capacities and Characterization of Compressible Clayey and Silty Foundation Soils,” 1980. [Online]. Available: https://shareok.org/handle/11244/316269. [Accessed: 22-Jun-2021].

[26] M. G. Stewart and D. V. Rosowsky, “Time-dependent reliability of deteriorating reinforced concrete bridge decks,” *Struct. Saf.*, vol. 20, no. 1, 1998.

[27] M. P. Enright and D. M. Frangopol, “Probabilistic analysis of resistance degradation of reinforced concrete bridge beams under corrosion,” *Eng. Struct.*, vol. 20, no. 11, 1998.
